# Clinical significance of matrix metalloproteinase-9 in Fragile X Syndrome

**DOI:** 10.1038/s41598-022-19476-y

**Published:** 2022-09-13

**Authors:** Asma Laroui, Luc Galarneau, Armita Abolghasemi, Sérine Benachenhou, Rosalie Plantefève, Fatima Zahra Bouchouirab, Jean François Lepage, François Corbin, Artuela Çaku

**Affiliations:** 1 Department of Biochemistry and Functional Genomics, Université de Sherbrooke, Sherbrooke, QC Canada; 2 Department of Medical Physics, McGill University Health Center, Montreal, QC Canada; 3 Department of Paediatrics, Université de Sherbrooke, Sherbrooke, QC Canada

**Keywords:** Analytical biochemistry, Biochemistry, Diseases

## Abstract

High plasma matrix metalloproteases-9 (MMP-9) levels have been reported in Fragile X Syndrome in a limited number of animal and human studies. Since the results obtained are method-dependent and not directly comparable, the clinical utility of MMP-9 measurement in FXS remains unclear. This study aimed to compare quantitative gel zymography and ELISA and to determine which method better discriminates abnormal MMP-9 levels of individuals with FXS from healthy controls and correlates with the clinical profile. The active and total forms of MMP-9 were quantified respectively, by gel zymography and ELISA in a cohort of FXS (*n* = 23) and healthy controls (*n* = 20). The clinical profile was assessed for the FXS group using the Aberrant Behavior Checklist FXS adapted version (ABC-C_FX_), Adaptive Behavior Assessment System (ABAS), Social Communication Questionnaire (SCQ), and Anxiety Depression and Mood Scale questionnaires. Method comparison showed a disagreement between gel zymography and ELISA with a constant error of − 0.18 [95% CI: − 0.35 to − 0.02] and a proportional error of 2.31 [95% CI: 1.53 to 3.24]. Plasma level of MMP-9 active form was significantly higher in FXS (*n* = 12) as compared to their age-sex and BMI matched controls (*n* = 12) (*p* = 0.039) and correlated with ABC-C_FX_ (r_*s*_ = 0.60; *p* = 0.039) and ADAMS (r_*s*_ = 0.57; *p* = 0.043) scores. As compared to the plasma total form, the plasma MMP-9 active form better enables the discrimination of individuals with FXS from controls and correlates with the clinical profile. Our results highlight the importance of choosing the appropriate method to quantify plasma MMP-9 in future FXS clinical studies.

## Introduction

Fragile X Syndrome (FXS) is the most common monogenic cause of autism spectrum disorder (ASD) and intellectual disability (ID)^1,2^. FXS is caused by mutations of the X-linked *Fragile X messenger ribonucleoprotein 1* (*FMR1*)^3,4^. In the majority of cases, it results from an abnormal trinucleotide repeat (CGG) in the promotor region of *FMR1*^5^ leading to a deficit of X messenger ribonucleoprotein, or FMRP. FMRP is a ubiquitous RNA-binding protein, highly expressed in the brain^6,7^, where it controls the translation of up to 4% of RNAs coding proteins^8^ essential for synaptic plasticity^9^. Hence, the absence of FMRP leads to a hyper-synthesis of dendritic proteins resulting in immature, thin, and long dendritic spines, which are in part responsible for the neurodevelopmental impairment of FXS^10–12^.

Matrix metalloproteinase 9 (MMP-9), a member of the MMPs family, is a dendritic protein whose expression is tightly regulated by FMRP in neurons^13^. Specifically, upon synaptic stimulation of the glutamate receptor (mGluR), FMRP dissociates from the MMP-9 mRNA complex allowing MMP-9 translation to occur^14^. In FXS, the absence of FMRP leads to excessive synthesis of MMP-9. Studies in *Fmr1* knock‐out (KO) mice highlight the implication of MMP-9 in the FXS pathophysiology^13–15^. Indeed, high MMP-9 levels have been reported in brain tissue and plasma of FXS animal models and humans. Moreover, treatment with minocycline (a tetracycline derivative) an inhibitor of circulating metalloproteinases, reduced MMP-9 activity, restored the synaptic development in *Fmr1* KO mice, and improved aberrant behavior of *Fmr1* KO mice and FXS patients^16–18^.

MMP-9 is synthesized as an inactive proenzyme (pro-MMP-9, 92 kD) that requires removal of the pro-domain to achieve the active form (MMP-9, 87 kD). The latter is a Zn^2+^ dependent endopeptidase that degrades collagen type IV substrate^19,20^. Different methods have been applied to quantify MMP-9 total (pro and active form) and active forms in brain tissues or plasma. Specifically, gelatin-based zymography^16,21^ measures measure both, the pro-MMP-9 and the active forms. Whereas, Enzyme-Linked Immunosorbent Assays (ELISA)^14^ and Western Blot^22^ measure the total form.

Studies in *Fmr1* KO mice had shown an increase of both, total and active MMP-9 forms in the brain^15,22,23^. To the best of our knowledge, only two studies focused on MMP-9 measurements in individuals with FXS. The first showed an increase of MMP-9 total form in post-mortem brains (measured by Western blotting) in eight individuals with FXS as compared to nine controls^22^. The second study revealed an increase of plasma MMP-9 active form (as measured by semiquantitative gel zymography) of ten individuals with FXS as compared to eight age and sex-matched controls^16^. Moreover, plasma MMP-9 has been suggested as a potential biomarker to monitor outcomes of clinical trial in FXS^24,25^. To the best of our knowledge, no study compared the clinical significance of plasma total versus active MMP-9 forms in FXS.

The present work focused on the quantification of plasma total and active MMP-9 forms using, respectively, ELISA and gel zymography in individuals with FXS and healthy controls. Our objectives were to compare the two methods in order to determine which MMP-9 plasma form (total vs. active form) enables the best discrimination of individuals with FXS from controls and correlates better to the clinical profile.

## Materials and methods

### Study population

The study population is a sub-cohort of the LipidX study comprising 23 individuals with FXS(diagnosis confirmed by southern blot and polymerase chain reaction) and 20 healthy controls^26^. FXS participants were recruited at the FXS clinic located at CIUSSS de l’Estrie-CHUS, while controls were recruited via local community. Admissibility criteria were previously reported^26^. All participants gave their written informed consent before participating in the study. The project was performed according to the protocol approved by the Ethics Board of the Research Center of *the Centre Hospitalier Universitaire de Sherbrooke*, Canada (Project number 2020–3552). The study was performed in accordance with the Declaration of Helsinki. Blood samples were collected in K_2_EDTA tubes (BD Vacutainer^®^). Plasma was recovered following successive centrifugations (300 X g for 10 min and 2400 × g for 15 min to remove all cellular components) and stored at − 20 °C. FMRP was performed on whole cell extracts from platelets (pg/10^6^ platelets), by Western blot as previously prescribed^27^.

### Gel zymography

The gel zymography protocol has been previously described^21,28^. Briefly**, **1 µl of plasma was diluted in Laemmli buffer and loaded on an 8% polyacrylamide gel (SDS-PAGE) containing 2 mg/ml gelatin. Following electrophoretic separation, gels were washed twice for 30 min in 2.5% Triton X-100, 50 mM Tris-HCL-PH 7.5, 5 mM CaCl_2 _and 1 µM ZnCl_2_ and then incubated for 18 h at 37 °C in 50 mM Tris-HCl-pH 7.5, 5 mM CaCl_2_, 1 µM ZnCl_2_, 1% Triton X100 buffer, with gentle agitation. Proteins were stained with Coomassie Brilliant Blue in 40% methanol for 45 min and distained in 40% methanol and 10% acetic acid for 30 min. The gels were scanned on a BIORAD; ChemiDoc™ MP Imaging System. Areas of enzyme activity (clear bands over the blue dark background) were quantified in Image J software^29^. Only the active form of MMP-9 at 82 kD (identified with molecular weight standards) was quantified. The recombinant human MMP-9 standard (Abcam^©^ Inc: Natural human MMP-9 protein: proenzyme monomer, N° 157,344) was first activated with 1 mM 4-aminophenylmercuric acetate (APMA) and then used for quantification as calibrator in a standard curve within the range of 100 pg/well to 600 pg/well. Samples were quantified in duplicates and a positive plasma control was carried on every gel to monitor the reproducibility of the assay.

### Immunoassay ELISA

ELISA immunoassays were performed according to the manufacturer's instruction (R&D systems) (Catalog#DY911-05). Briefly, the standard curve was obtained using serial dilutions of recombinant human MMP-9 (quantification range: 31.3 to 2000 pg/ml). Plasma samples were diluted 1/1000 in phosphate buffered saline (PBS) containing 1% bovine serum albumin (BSA).

### Clinical profile

The clinical profile was assessed using the following questionnaires: Aberrant Behavior Checklist-Community (ABC-C), Adaptive Behavior Assessment System (ABAS), Social Communication Questionnaire (SCQ), and Anxiety Depression and Mood Scale (ADAMS).

The ABC-C was developed to measure aberrant behavioral among individuals with ID^30^. This questionnaire has been validated in the FXS population as a six-factor structure known as the FXS version of ABC-C (ABC-C_FX_)^31^. It uses a 58-items rating scale evaluating six behavior domains: irritability, hyperactivity, social unresponsive, social avoidance, stereotypy, and inappropriate speech. Each item is rated from 0 to 3 as follow: 0 (not at all a problem); 1 (slight problem); 2 (moderately serious problem); 3 (severe problem).

The ABAS-II questionnaire evaluates adaptive behavior and skills, categorised in three domains including conceptual, social, and practical. Each item is scored as follow: 0 (not able); 1 (never or rarely); 2 (sometimes) and 3 (always or almost always)^32^.

The SCQ is a screening questionnaire for ASD composed of 40 questions (yes/no) evaluating language, communication, reciprocal social interaction, stereotyped and repetitive patterns of behavior^33^.

The ADAMS questionnaire is used to assess behavior-based affective symptoms of individuals with ID older than 10 years old. It consists of 29 items clustered into five subscales: hyperactive behavior, depressed mood, social avoidance general anxiety, and obsessive/compulsive behavior.

Each item is scored from 0 (behavior has not occurred or is not a problem) to 3 (behavior occurs a lot or is a severe problem)^34^.

### Statistical analysis

All statistics were performed in GraphPad Prism 8. For the ELISA assay, the concentration of MMP-9 was calculated using a four-parameter logistic function (4-PL). The distribution of each data was tested by D'Agostino & Pearson test and the Shapiro–Wilk test. Deming (Model II) linear regression was used to compare the two analysis methods and the Bland–Altman (BA) to compare the measurements of the same variable by both methods. Spearman’s rank correlation was performed to evaluate the association between MMP-9 activity and the clinical profile. A false discovery (FDR) adjusted *p*-value of 0.05 was used as a statistical significance threshold.

### Ethics approval and consent to participate

The project was approved by the Ethics Board of the Research Center of *the Centre Hospitalier Universitaire de Sherbrooke*, Canada. All participants gave their written informed consent before participating in the study.

## Results

### Study population

Our study population included 23 individuals with FXS and 20 healthy controls. Males represented the majority of both groups with a higher representation in FXS than in controls (87% vs. 57%). 14 individuals with FXS were under medication, with 10 of them taking at least 2 medications. The latter included: antipsychotics, selective serotonin reuptake inhibitors and stimulants. None of the participants experienced or reported an acute illness. The FXS group had a higher body mass index (BMI) as compared to controls (26 vs. 21.9 kg/m^2^). In a subgroup of 12 FXS participants matched for age and sex with 12 healthy controls, the BMI was not significantly different between the groups. Characteristics of participants are shown in Table 1.

**Table 1:** Characteristics of participants.

	FXS	Controls	*p*-value
All (n)	23	20	
Age in years, median (range)	24 (17– 41)	22 (20–41)	0.776**
**Males n (%)**	21 (91)	12 (60)	0.03*
Full mutation (*n*)	17		
Mosaic (*n*)	4		
Individuals expressing FMRP (%)	35	100	< 0.01*
BMI Kg/m^2^ median (range)	26.0 (19.2–41.4)	21.9 (18.7–32.8)	0.01**
**Comorbidities (%)**			
Attention deficit hyperactivity disorder	26.09		
Anxiety	39.13		
Epilepsy	13.04		
Other (neurology, endocrinology, and nephrology)	21.71		
**Matched participants (*n*)**	12	12	
Age, median (range)	24 (17–28)	22 (20–29)	0.808**
Full mutation (*n*)	11		
Mosaic (*n*)	1		
BMI Kg/m^2^ median (range)	25.5 (19.4–39.1)	23.3 (18.7–32.8)	0.259**

**Mann–Whitney test.

*Fisher exact test.

### Method comparison

**Figure 1:**
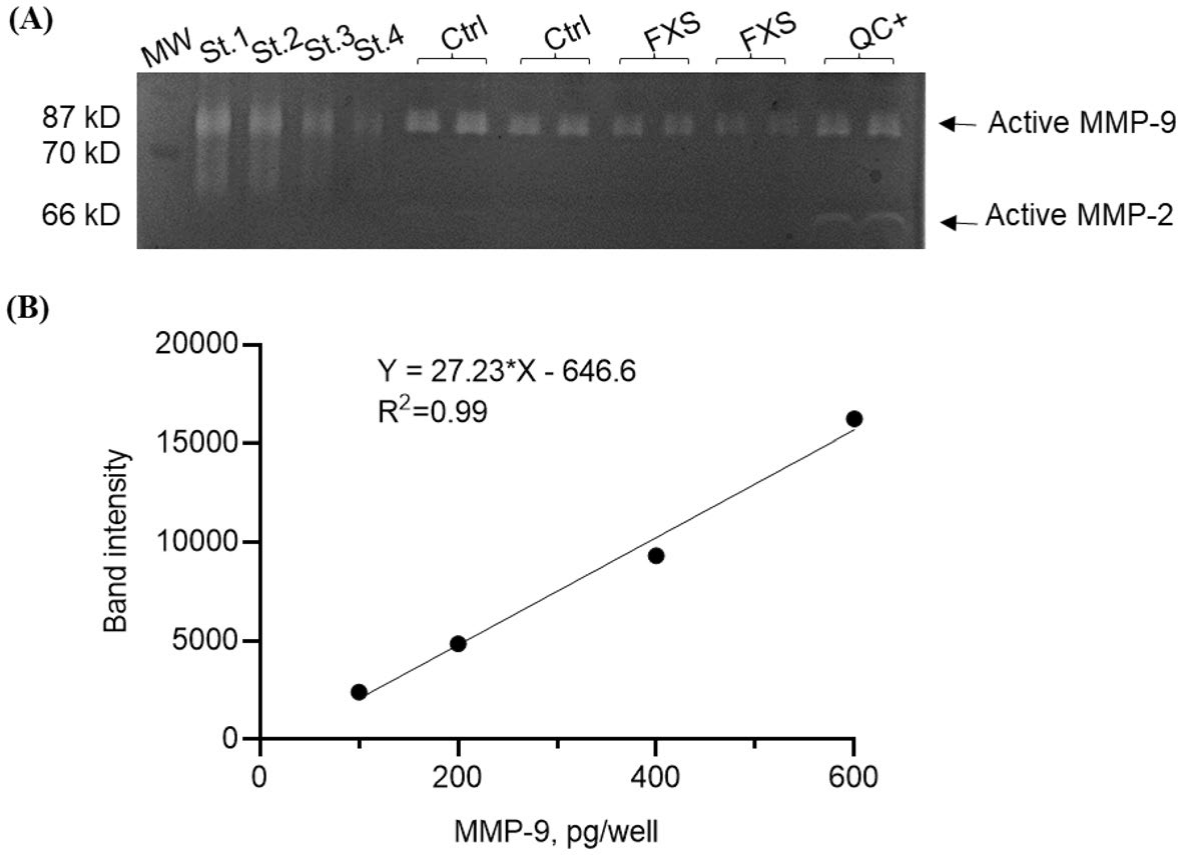
Gel Zymography quantification. (**A**) A representative separation of active MMP-9 and MMP-2 by gel zymography. St.1 to St.4 correspond to standard recombinant human MMP-9 used as calibrator; Ctrl and FXS are samples from controls and FXS participants, in duplicate; QC + represents the positive quality control. (**B**) A typical standard curve of active MMP-9 obtained by gel zymography.

Based on the ability of MMP-9 and MMP-2 to degrade gelatin, gel zymography allowed the identification of their active forms (Fig. 1A). Only the active form of MMP-9 (87 KD) was quantified in the present study. The linearity of the standard curve for gel zymography obtained within a range of 100 to 600 pg of MMP-9 per well (corresponding to plasma concentration ranging from 0.1 to 0.6 mg/l) showed consistently a R^2 ^> 0.99 (Fig. 1B). The linearity of the standard curve for ELISA obtained within a range of 31.3 to 2000 pg/ml (corresponding to plasma concentration starting from 0.03 to 2 mg/l) consistently show a R^2^ > 0.99. For both methods, the same plasma pool was run as a positive quality control (QC^+^). The intra-assay and inter-assay coefficient of variation (CV) were lower than 20%.

**Figure 2:**
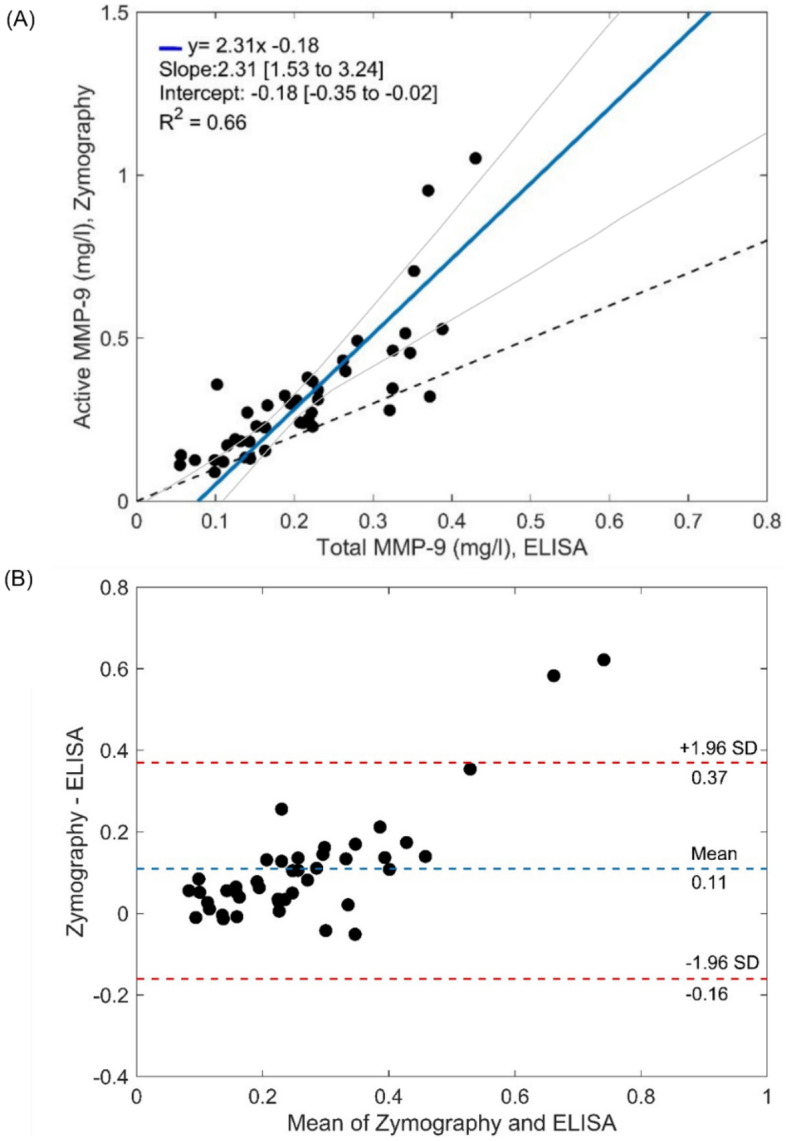
Comparison of two MMP-9 quantification methods (gel Zymography and ELISA) in 43 individuals. (**A**) Deming regression (blue line) with confidence interval (gray lines); black dashed line represents ideal correlation (*Y* = *X)*. (**B**) Associated Bland Altman plots. The limits of agreement are defined as the mean differences ± 1.96 SD of differences and is shown in red dashed lines. Samples were run in duplicate.

To compare results obtained by gel zymography and ELISA, we first performed a Pearson correlation test. Since the Pearson’s coefficient *r* was lower than 0.97 (*r* = 0.81), the Deming’s (Model II) regression was applied to test the agreement between the two methods. Our results showed a statistically significant proportional and constant difference between the two methods, respectively, with a slope coefficient of 2.31 (confidential interval: 1.53 to 3.24; *p* < 0.001) and an intercept value of − 0.18 (Confidential interval: − 0.35 to − 0.02) (Fig. 2A).

In addition, the Bland–Altman difference plot confirmed the presence of a positive bias of 0.11 mg/l between zymography and ELISA results, with a disagreement between the two methods at concentrations higher than 0.5 mg/l (Fig. 2B).

### Active and total MMP-9 plasma levels in FXS and healthy controls

**Figure 3:**
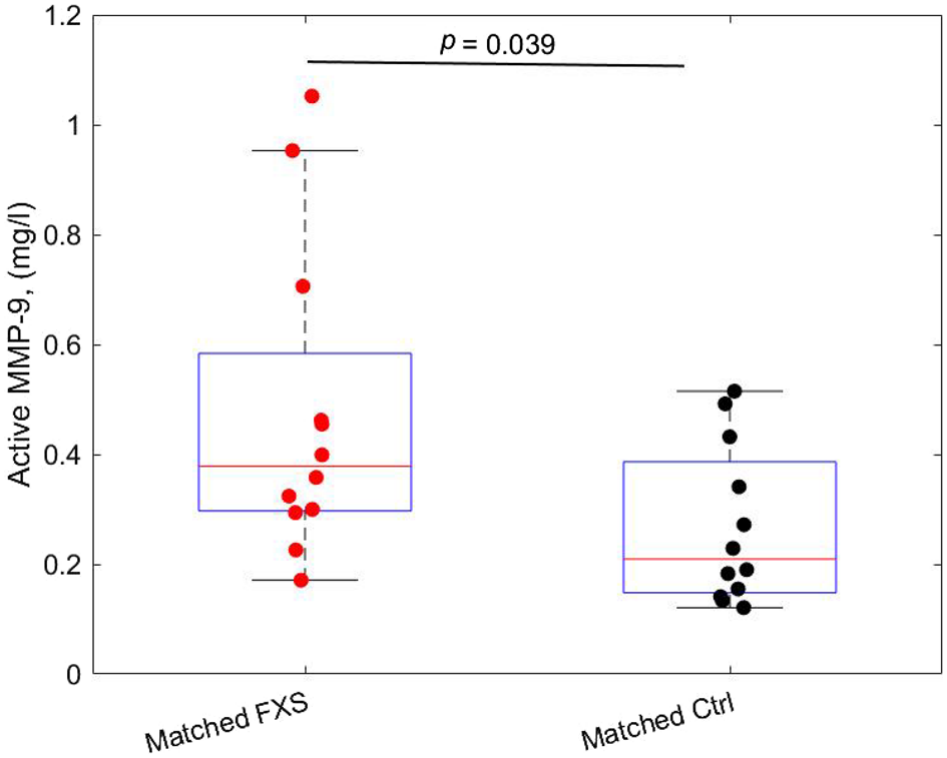
Plasma MMP-9 active measured by gel zymography in 12 FXS participants and 12 age-sex and BMI matched controls. Samples were run in duplicate.

Active and total MMP-9 plasma levels were first compared for all study participants and no significant difference was obtained between FXS and controls (Supplemental Table 1). Since MMP-9 plasma levels are influenced by age^35^, sex^36^ and BMI^37^, the comparison was then performed in a subgroup of 12 FXS and 12 controls matched for all these variables. The concentration of plasma MMP-9 active form as measured by gel zymography was higher in the FXS group as compared to controls (0.48 mg/l ± 0.28 vs. 0.27 mg/l ± 0.14; *p* < 0.05) (Fig. 3), while plasma total MMP-9 level assessed by ELISA showed no statically significant difference (Supplemental Table 1).

### MMP-9 and clinical profile of individuals with FXS

The plasma MMP-9 active form showed a positive association with the aberrant behavior of FXS participants as evaluated by the ABC-C_FX_ 's total score (*r*_*s*_ = 0.60, *p* = 0.039).

Specifically, a significant correlation was obtained for four over six ABC-C_FX_ sub-domains including hyperactivity (*r*_*s*_ = 0.55, *p* = 0.033), social unresponsive (*r*_*s*_ = 0.73, *p* = 0.003), stereotypy (*r*_*s*_ = 0.51, *p* = 0.019) and inappropriate speech (*r*_*s*_ = 0.55, *p* = 0.040). Moreover, the level of plasma MMP-9 active form significantly correlated with the ADAMS score (*r*_*s*_ = 0.57, *p* = 0.043) (Table 2). On the other hand, plasma MMP-9 total form measured by ELISA showed a significant association only with the social unresponsive ABC-C_FX_ subdomain (Table 2). No significant association was observed with ABAS or SCQ scores for neither the active nor the total MMP-9 forms (Supplemental Table 2).

**Table 2:** Association of plasma MMP-9 forms with clinical profile of individuals with FXS.

*n* = 23	Zymography		ELISA	
	*r*_s_	*p*	*r*_s_	*p*
ABC-C_FX_ subscales				
Irritability	0.21	0.579	0.07	1.033
Hyperactivity	0.55	**0.033**	0.32	0.267
Social unresponsive	0.73	**0.003**	0.56	**0.040**
Social avoidance	0.41	0.155	0.31	0.284
Stereotypy	0.51	**0.019**	0.36	0.192
Inappropriate speech	0.55	**0.040**	0.39	0.087
Total	0.60	**0.039**	0.41	0.159
ADAMS	0.57	**0.043**	0.38	0.192

*r*_*s*_: Spearman’s Rho; *p*: *FDR*- adjusted *p*-value for multiple testing.

Significant values are in bold.

## Discussion

Here we report the first study comparing two quantification methods of plasma MMP-9 in FXS. From the clinical endpoint, the MMP-9 active form better discriminates individuals with FXS from healthy controls and better correlates with the clinical profile as compared to the MMP-9 total form.

As in previous reports, we found a strong correlation (*r* = 0.81) between MMP-9 active and total form measured, respectively, by gel zymography and ELISA^38,39^. However, the Pearson’s correlation coefficient (*r*) does not assess agreement between the two methods but rather measures the association between them. Therefore, it is not adequate to use the correlation as a single metric for method comparison studies^40^. According to CLSI guidelines^41^ when the correlation coefficient is greater than 0.975, simple linear regressions could be used to further explore constant and proportional errors; otherwise, more rigorous analyses such as Deming or Passing-Bablok regressions are needed^42^. Numerous studies quantified plasma MMP-9 in different pathologies, by gel zymography along with ELISA^43–46^. Heo et al. focused on method comparison of gel zymography and ELISA to ensure the reliability of the measurements and their results were based only on the coefficient of correlation^39^. To our knowledge, only one study performed additional statistical analyses. Specifically, Prescimone et al., compared both methods in 25 volunteers using correlation coefficient and Bland–Altman difference analysis. Based on the correlation coefficient and regardless of the absolute systematic error (0.006 mg/l) revealed by Bland–Altman analysis, they concluded that both methods offer similar reliability for quantifying MMP-9^38^. In accordance with their results, we obtained a positive bias between gel zymography and ELISA, but on a significant larger scale (0.11 mg/l). The discrepancy between the bias results might be explained by distinct MMP-9 standards used as calibrators for gel zymography and/or ELISA kits; the variation of ranges of measure (0.00025–0.001 mg/l vs. 0.1–0.6 mg/l of our cohort) and the difference in samples size (25 vs. 43 participants of our cohort).

We should note that Bland–Altman analysis is used to evaluate the acceptability based on the analytical imprecision of both methods, yet, to test whether a proportional error exists, a regression analysis must be applied. Considering a Pearson’s correlation coefficient of 0.81, we also performed Deming regression analysis. Our results revealed the presence of statistically significant systematic and proportional errors between the two methods. Indeed, circulating MMP-9 has different forms: the active and total MMP-9. Although their concentrations measured respectively by gel zymography and ELISA correlate between them, they do not represent the same biomarker. As such, finding that they differentially associate with clinical outcomes is unsurprising. Moreover, the present study includes the minimum number of samples (> 40 samples) required for a proper comparison of two biochemical methods^40^.

Here, we explored the clinical implication of different circulating forms of MMP-9 by questioning: first, the difference in total and active forms between FXS and controls; second, the association of specific MMP-9 forms with the clinical profile. No significant difference was observed between FXS and control individuals for neither active nor total form in the whole study population. However, considering changes in MMP-9 plasma level as a function of age^35,47^, sex^36,48^, and BMI^37,49^, we measured MMP-9 in a subgroup of 12 FXS and 12 controls matched for all these variables. Plasma levels of active MMP-9 obtained by gel zymography were significantly higher in FXS as compared to matched controls. These results are in agreement with a previous study^16^ showing an increase in plasma MMP-9 active form (semi-quantitatively determined by gel zymography) in ten individuals with FXS as compared to eight age-sex matched controls. In this study, we observed no significant difference in MMP-9 total form in FXS suggesting a better sensibility of gel zymography to distinguish FXS from control, based on MMP-9 active form.

Associations were observed between MMP-9 active form and several clinical features related to aberrant behavior, such as hyperactivity, social unresponsive, stereotypy, and inappropriate speech. Indeed, interventional trials in mice and humans with minocycline, a tetracycline derivative and an inhibitor of MMP-9 activity support these observations. Specifically, the minocycline treatment lowered MMP-9 active form, restored dendritic spine morphology^15^, and improved behavior performance of *Fmr1* KO mice^50^. Moreover, minocycline treatment was associated with a decrease of plasma MMP-9 active form^16^ and behavior improvement of FXS in humans including hyperactivity, stereotypy, inappropriate speech^18^.

Moreover, a direct association was also observed between the MMP-9 active form and the ADAMS score. Interestingly, anxiety is among neuropsychiatric FXS associated symptoms^34,51–53^ and inhibition of MMP-9 activity by minocycline showed an improvement of anxiety in individuals with FXS^18,54^. No significant association was obtained between MMP-9 plasma active or total forms and ABAS and SCQ scores suggesting that upregulation of MMP-9 active form might rather contribute to FXS-associated aberrant behavior and mood profile than to the cognitive dysfunction.

MMP-9 mRNA translation into MMP-9 is controlled by FMRP^23^. The proteolytic activation of MMP-9 which is required for synaptic activity is tightly regulated^14,50^. The up or down regulation of MMP-9 activity has detrimental effects on brain function^13^ and underlies deficits associated with many neurodevelopmental disorders such as autism spectrum disorder^55^. Indeed increased levels of MMP-9 have been reported in the amniotic fluid^55^ and in the serum of the patients with ASD^56^. The experimental data in ASD without FXS remains very limited as compared to ASD with FXS. Specifically, genetic removal of MMP-9 rescued the ASD-like symptoms of Fragile X in *Fmr1* KO mice^14^. In FXS, the deficit of FMRP leads to an uncontrolled upregulation of MMP-9 synthesis and consecutive increase of MMP-9 active form^23^. However, the underlining mechanism remains unknown and requires further investigation.

The results of current study should be considered in the light of some limitations. The sample size of matched groups was limited, therefore a larger number of FXS and matched controls are warranted to confirm the superiority of gel zymography utility over ELISA in FXS. In addition, both methods do not provide an accurate determination of plasma MMP-9 levels, but rather an estimation of MMP-9 activity (zymography) and total MMP-9 (ELISA). The MMP-9 regulating factors such as Tissue Inhibitor of Metalloproteinases (TIMPs) dissociate from MMP-9 during electrophoresis, therefore gel zymography might not give a precise measure of in vivo activity. Moreover, ELISA antibodies might cross react with TIMP-1 and thus preclude an accurate determination of the total form.The determination of the abundance of individual inhibitors would have provided an estimation of the degree of occupancy of MMP-9 by those inhibitors and thus a better measure of total MMP-9. However, the correction of the MMP-9 total form as measured by ELISA is theoretically needed but technically very challenging to obtain with today's methods. Furthermore, we used a manual technique to quantify the active form. Indeed, gel zymography is a time-consuming method that can be challenging to be performed properly and needs a well-trained technician. Although we considered the analytical variation of the methods and all experiments were performed by the same well-trained master student, other techniques providing optimal sensitivities and specificities, such as mass spectrometry could also be considered for the quantification of MMP-9 forms in a clinical context.

In conclusion, our results highlight the importance of choosing the appropriate method to quantify plasma MMP-9 in FXS studies, specifically in future clinical trials where MMP-9 might be used as a measure of outcomes^57^. Technically, ELISA is generally easier to perform than gel zymography and is more adaptable to upscale. However, gel zymography seems to be a more sensitive method to detect differences in MMP-9 active form between FXS and healthy controls as well as the association with aberrant behavior, and anxiety related symptoms.

## Supplementary Information

Supplementary Information.

## Data Availability

All data generated and analysed are included in this article.
