# Supplementary material for: Clinical significance of matrix metalloproteinase-9 in Fragile X Syndrome
Source: Sci Rep. 2022 Sep 13;12:15386. doi: 10.1038/s41598-022-19476-y (PMC9470743; doi:10.1038/s41598-022-19476-y)
Supplement: Supplementary file 1 — Supplementary Information. [file 41598_2022_19476_MOESM1_ESM.pdf]

Clinical significance of matrix metalloproteinase-9 in Fragile X Syndrome

Asma Laroui [1], Luc Galarneau [2], Armita Abolghasemi [1], Sérine Benachenhou[1], Rosalie Plantefève[1], Fatima Zahra Bouchouirab [1], Jean François Lepage[3], François Corbin[1] and Artuela Çaku[1]*

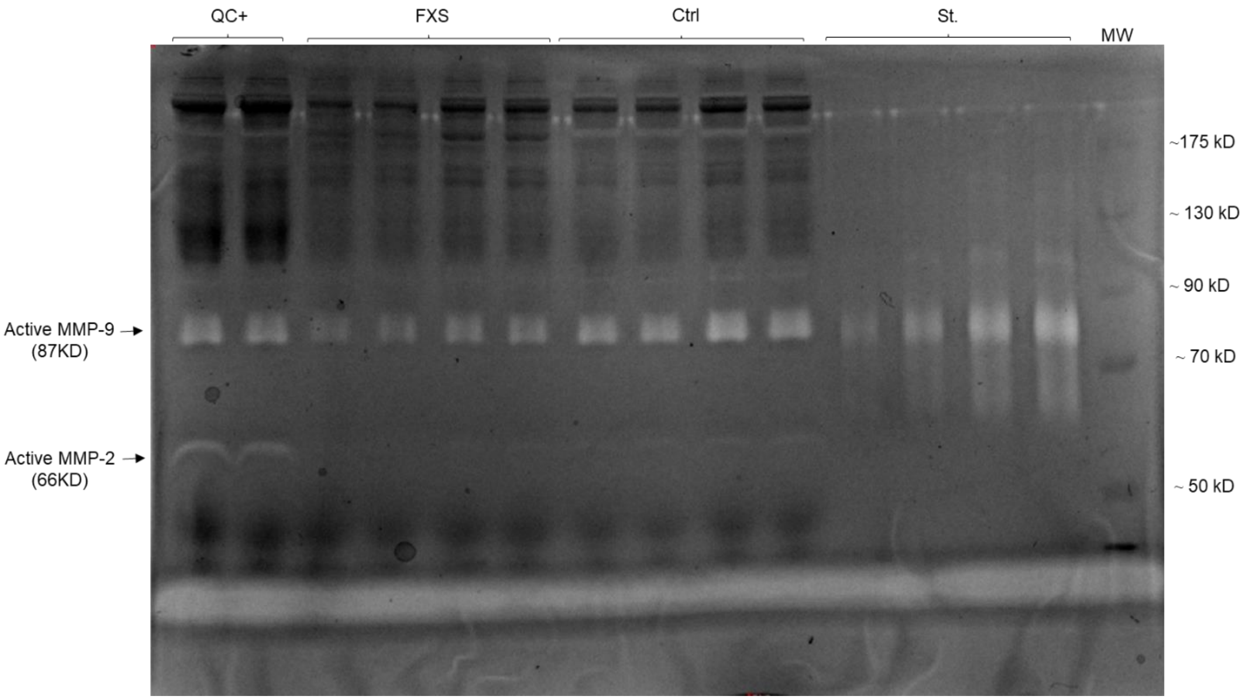

Supplemental Figure 1. Gel Zymography quantification. A representative separation of active MMP-9 and MMP-2 by gel zymography. St. corresponds to standard recombinant human MMP-9 used as calibrator; Ctrl and FXS are samples from controls and FXS participants, in duplicate; QC+ represents the positive quality control.

Clinical significance of matrix metalloproteinase-9 in Fragile X Syndrome

Asma Laroui [1], Luc Galarneau [2], Armita Abolghasemi [1], Sérine Benachenhou[1], Rosalie Plantefève[1], Fatima Zahra Bouchouirab [1], Jean François Lepage[3], François Corbin[1] and Artuela Çaku[1]*

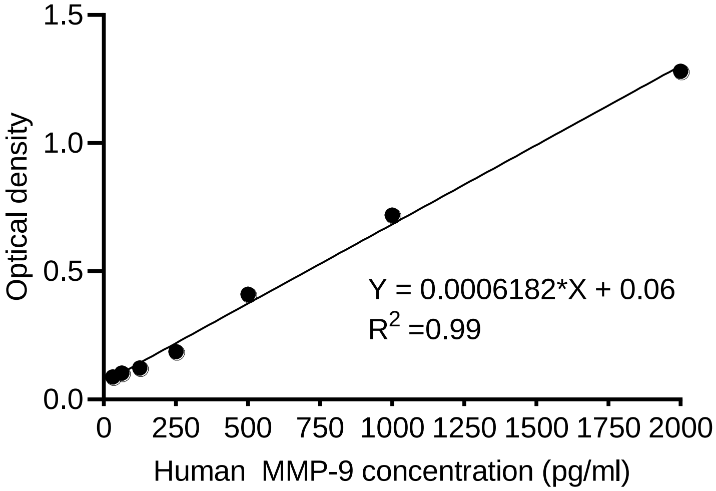

Supplemental Figure 2. Total MMP-9 standard curve obtained by ELISA.

Clinical significance of matrix metalloproteinase-9 in Fragile X Syndrome

Asma Laroui [1], Luc Galarneau [2], Armita Abolghasemi [1], Sérine Benachenhou[1], Rosalie Plantefève[1], Fatima Zahra Bouchouirab [1], Jean François Lepage[3], François Corbin[1] and Artuela Çaku[1]*

Supplemental Table 1. Active and total MMP-9 plasma levels in FXS and healthy controls. Samples were run in duplicate.

	Active MMP-9	Total MMP-9
All FXS (*n*= 23)	0.37 mg/l ± 0.25	0.22 mg/l ± 0.11
All controls (*n*= 20)	0.26 mg/l ± 0.11	0.20 mg/l ± 0.08
P-value *	0.193	0.796
Matched FXS (*n*= 12)	0.48 mg/l ± 0.28	0.25 mg/l ± 0.11
Matched controls (*n*=12)	0.27 mg/l ± 0.14	0.18 mg/l ± 0.08
P-value *	0.039 *	0.117

The table represents Mean values ± SD.

 * *Mann Whitney U test.*

Supplemental Table 2. Association of plasma MMP-9 forms with clinical profile of FXS individuals.

	**Zymography**		**ELISA**	
n=23	r_s	*p*	r_s	*p*
FXS edition ABC-C subscales				
Irritability	0.21	0.579	0.07	1.033
Hyperactivity	0.55	**0.033**	0.32	0.267
Social unresponsive	0.73	**0.003**	0.56	**0.040**
Social avoidance	0.41	0,155	0.31	0.284
Stereotypy	0.51	**0,019**	0.36	0.192
Inappropriate speech	0.55	**0.040**	0.39	0.087
Total	0.60	**0.039**	0.41	0.159
ADAMS	0.57	**0.043**	0.38	0.192
ABAS-II				
Conceptual	-0.4606	0.132	-0.3183	0.189
Social	-0.1988	0.593	-0.08175	0.725
Practical	-0.4259	0.176	-0.3119	0.191
Global	-0.38	0.203	-0.2048	0.274
SCQ	-0.1369	0.816	-0.2048	0.402

r_{S}: *Spearman's Rho*; *p*: *FDR- adjusted p-value for multiple testing.*
